# S09-1: The Experiences of People with Disabilities in Sport & Physical Activity

**DOI:** 10.1093/eurpub/ckae114.236

**Published:** 2024-09-26

**Authors:** Robert Purcell

**Affiliations:** Sport Ireland, Dublin, Ireland; Active Disability Ireland, Ireland

## Abstract

**Purpose:**

In April 2022, over 1.1 million people reported having experienced at least one long-lasting condition or disability (Census, 2022). Research has highlighted that people with disabilities in Ireland are far less likely to be active than those without a disability with a difference of 28% vs 48% playing sport on a regular basis (Irish Sports Monitor (ISM), 2022). This difference is also apparent in children, the Children’s Sport Participation and Physical Activity Study (CSPPA, 2022) reported that primary school children with disabilities are less likely to meet physical activity guidelines than those without a disability (15% vs 24%). Additionally, there were significantly lower rates of overall community and school sport participation amongst students with disabilities (primary: 92%, post-primary: 77%) compared to those without disabilities (primary: 97%; post-primary: 87%).

**Methods:**

To understand this difference, Active Disability Ireland undertook research to explore the lived experience of youths with disabilities, and their parents/guardians, regarding physical activity. The consultation involved a phenomenological approach utilising a mixed methodology of surveys and semi-structured interviews with the goal of opening discussion spaces for youths with disabilities and their guardians to share their experiences, challenges and needs in leading active and healthy lifestyles. Participants were invited to be part of a youth forum to continue providing valuable insights.

**Results:**

Our results suggest that for young people with disabilities, physical activity is seen as particularly important as well as enjoyable. However, only 27 % agreed that they find it easy to take part in physical activity. A lack of opportunities, a lack of self-confidence and having no-one to take part with were commonly cited as major barriers to participation. Parents/guardians also expressed difficulties such as a lack of suitable places or facilities, a lack of support/understanding from people working in the sector and a worry about their child not being able to take part.

**Conclusions:**

Programmes and policies to address the barriers found in this study may address current inequalities and contribute to reducing the disability gradient in sports participation.

**Support/Funding Source:**

Active Disability Ireland, Sport Ireland

